# CAMEO: a CAZyme mapping engine optimized for HUMAnN

**DOI:** 10.1128/mra.00707-26

**Published:** 2026-07-31

**Authors:** Matthew J. Bolino, Steven A. Frese

**Affiliations:** 1 Department of Nutrition, University of Nevada 6851, Reno, Nevada, USA; 2 University of Nevada, Reno School of Medicine 12290, Reno, Nevada, USA; University of Michigan, Ann Arbor, Michigan, USA

**Keywords:** metagenomics, HUMAnN, bioinformatics, CAZyme, carbohydrates, gut microbiome

## Abstract

There are technical barriers to creating functional mapping databases and a dearth of validated databases that can be easily implemented by users. We present CAMEO, a precomputed and validated mapping file for carbohydrate-active enzymes, as well as an approach to building new CAZyme mapping files, for use with HUMAnN.

## ANNOUNCEMENT

The increasing interest in and accessibility of metagenomic and meta-transcriptomic applications have been facilitated by the development of software tools, such as bioBakery (1). Metagenome-assembled genomes (MAGs) are popular (2–5), but short read mapping is a strong and efficient alternative. HUMAnN, part of the bioBakery, maps short reads for functional analysis using a reference database (6). Accessory tools within HUMAnN then allow for features to be regrouped against various functional pathways or annotations using included mapping files. However, there are limited options for robust and efficient mapping with other annotations, such as Carbohydrate-Active Enzymes (CAZymes).

**Fig 1:**
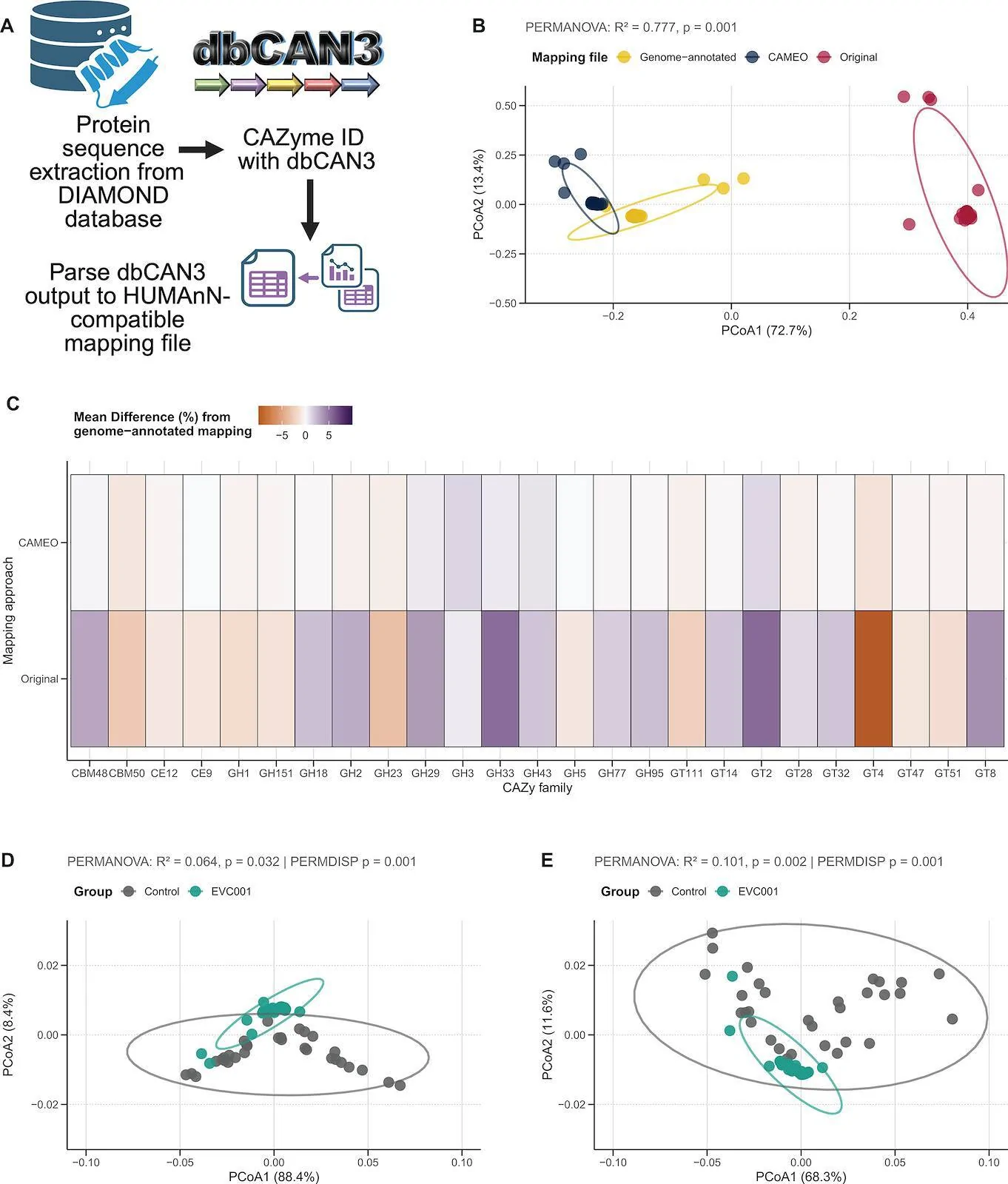
CAMEO generation workflow and CAZyme analysis. (**A**) A schematic of CAMEO generation using the HUMAnN DIAMOND database and dbCAN3. (**B**) A comparison of shotgun metagenomes from *Bifidobacterium longum* subsp. *infantis* EVC001*-*colonized infants (mean relative abundance ~88%, *N* = 29) mapped with either a genome-derived annotation for *B. infantis* CAZymes, the CAMEO-built mapping file, or a previous version of a CAZyme mapping file (“Original”). (**C**) Mean differences between the CAMEO-derived and the “original” mapping file relative to the genome-derived mapping file (“genome-annotated”) are shown, with values closer to zero indicating a stronger mapping performance. Here, samples with high levels of *B. infantis* (~88% mean abundance) appear to map more effectively with the CAMEO mapping file. (**D and E**) A principal coordinate plot (PCoA) comparing Bray-Curtis distances between EVC001-colonized (~88% *B*. *infantis* EVC001 relative abundance, *N* = 29) and control infant fecal samples (lacking *B. infantis*, *N* = 31) using the original (**D**) or CAMEO (**E**) mapping strategies, respectively. While the overall relationships between samples is generally preserved, we see more dispersal among control samples (gray) in the CAMEO-mapped figure and a higher *R*^2^ result from the PERMANOVA.

To help address this, we developed CAMEO: a CAZyme mapping engine optimized for HUMAnN. CAMEO was generated by extracting protein sequences directly from a UniRef90 database created with DIAMOND (7) (v2.1.16) for HUMAnN (v3.9), and then passing these sequences through dbCAN3 (8) (v3.0.6) to identify CAZymes. UniRef proteins identified as CAZymes by dbCAN3 were then parsed to create a HUMAnN-compatible mapping file, effectively connecting dbCAN3’s CAZyme predictions to the UniRef90 database in HUMAnN. While there are many tools to identify CAZymes, such as Cayman (9) and dbCAN (8), these tools require front-end sequence filtering, mapping, and/or read assembly separately from the read mapping performed by HUMAnN. This complicates interpretation and integration and limits their use. Here, we provide a precomputed CAZyme mapping file for HUMAnN compatible with the UniRef90 database (Fig. 1A). The workflow to re-calculate this mapping file using another database (e.g., UniRef50) or another function calling strategy (e.g., antibiotic resistance genes) is also provided. We further tested this new CAZyme mapping strategy in HUMAnN using fecal metagenomic sequencing data and found that CAMEO outperforms a previously circulated CAZyme mapping file for HUMAnN (10).

To validate this method, a shotgun metagenome data set from infants colonized with high levels by *Bifidobacterium longum* subsp. *infantis* EVC001 (*N* = 29) as well as a control group lacking this organism (*N* = 31) (11–13) were analyzed using a mapping file posted in the bioBakery forums in 2021 (10) created using the same approach as the built-in mapping files for HUMAnN3 (6, 10) (the “original”), a mapping file built from the *B. infantis* ATCC15697^T^ genome (14) with known CAZyme annotations (“genome-annotated”), and the CAMEO mapping file. Comparing results across these mapping strategies, the CAMEO- and genome-derived annotation strategies were most similar (Fig. 1B and C). When we compared the *B. infantis* EVC001-colonized samples to the control samples (Fig. 1D and E), we also found an improvement in CAZyme discrimination (*R*^2 =^ 0.064 vs. 0.101) over the original mapping file.

CAMEO provides an updated Uniref90-to-CAZyme HUMAnN-compatible mapping file that captures a broader CAZyme composition with better recovery compared to a previous mapping approach (10). To rebuild the mapping file for any database of interest, the necessary code is provided as shell and Python scripts, and future HUMAnN database releases will be used to update CAMEO. We have also included a software container to run the workflow across a variety of compute settings. CAMEO offers a better approach to assess microbial CAZyme composition with enhanced accuracy and read mapping for use with popular tools such as HUMAnN.

## Data Availability

The referenced mapping file and the code to compute the file are available at the project’s GitHub repository under the GNU GPL v3 license: github.com/koitaxoumemesa/cameo and at https://doi.org/10.5281/zenodo.21074017. The referenced metagenomic sequencing data are available in the NCBI SRA under accession number PRJNA390646.

## EDITOR'S NOTE

**Ed. Note**: This article was handled by Pat Schloss, who acted as a guest editor in consultation with the editor in chief, Irene Newton. Dr. Schloss's role as the guest editor of this article is outside of his duties as the chair of the Journals Committee.
